# Serum levels of matrix metalloproteinase 9 and toll-like receptor 4 in acute aortic dissection: a case-control study

**DOI:** 10.1186/s12872-018-0958-2

**Published:** 2018-11-29

**Authors:** Tan Li, Jing-Jing Jing, Jun Yang, Li-Ping Sun, Yue-Hua Gong, Shi-Jie Xin, Yuan Yuan

**Affiliations:** 1 Tumor Etiology and Screening Department of Cancer Institute and General Surgery, the First Hospital of China Medical University, and Key Laboratory of Cancer Etiology and Prevention (China Medical University), Liaoning Provincial Education Department, No.155 NanjingBei Street, Heping District, Shenyang, Liaoning Province 110001 China; 2 Department of Cardiovascular Ultrasound, the First Hospital of China Medical University, Shenyang, 110001 China; 3 Department of Vascular and Thyroid Surgery, the First Hospital of China Medical University, No.155 NanjingBei Street, Heping District, Shenyang, Liaoning Province 110001 China

**Keywords:** Matrix metalloproteinase 9, Toll-like receptor 4, Acute aortic dissection, Inflammation

## Abstract

**Background:**

Matrix metalloproteinase 9 (MMP9) and Toll-like receptor 4 (TLR4) play important roles in aortic pathophysiology. However, there is lacking research on serum TLR4 levels in acute aortic dissection (AAD) patients, and the performance of serum MMP9 and TLR4 for the diagnosis of AAD is still unknown. This study aimed to evaluate the serum levels of MMP9 and TLR4 in AAD patients, identify their associations with circulating C-reactive protein (CRP) and D-dimer, which are well-known classical biomarkers of AAD, and further explore the potential diagnostic role of MMP9 and TLR4 in AAD.

**Methods:**

Serum levels of MMP9 and TLR4 were measured by enzyme-linked immunosorbent assay (ELISA) in 88 AAD patients and 88 controls. The clinical test related information was collected from patients’ electronic medical records.

**Results:**

Serum MMP9 and TLR4 levels were significantly higher in AAD patients than those in healthy controls in the general and stratified comparisons. Either serum MMP9 or TLR4 was independently associated with the risk of AAD (all *p* < 0.001). There was a positive significant association between serum MMP9 and TLR4 (*r* = 0.518, *p* < 0.001). Both MMP9 and TLR4 levels were statistically correlated with circulating CRP, but not D-dimer. Based on receiver-operating characteristic (ROC) analysis, the area under the curves (AUCs) of MMP9 and TLR4 alone for the diagnosis of AAD were 0.810 and 0.799 with optimal cut-off points of 379.47 ng/ml and 7.83 ng/ml, respectively. Moreover, a combination of serum MMP9 and TLR4 increased the AUC to 0.89 with a sensitivity of 60.2% and specificity of 94.3%.

**Conclusions:**

Serum MMP9 and TLR4 could be potential biomarkers for identifying AAD, while the combined diagnostic value was higher in safely ruling out AAD.

## Background

Acute aortic dissection (AAD), a life-threatening aortic lesion, is characterized by the separation of aortic wall layers with severe morbidity and mortality. Based on the site of lesion and extended-range, AAD is most commonly divided in Stanford type A (involving the ascending aorta, proximal dissection) and Stanford type B (involving the descending aorta, distal dissection) [1]. The progressive extracellular matrix proteins degradation, and local aortic wall and systemic inflammatory reactions are regarded as key pathological features in AAD formation and development [2]. Matrix metalloproteinase 9 (MMP9) is an important extracellular matrix proteinase with the ability to degrade multiple extracellular components in aortic wall, mainly collagen and elastin, and its excessive production can result in the weakening of the aortic media, pathologic vascular remodeling and dissection [3]. MMP9 is produced by endothelial cells, smooth muscle cells, fibroblasts and infiltrating inflammatory cells [4]. Several studies have demonstrated that serum MMP9 levels measured by enzyme-linked immunosorbent assay (ELISA) in patients with AAD were higher than those in normal population [5–7]. However, the potential clinical diagnostic value of serum MMP9 in identifying AAD is far from clear.

Increasing data have indicated that AAD is closely associated with an inflammatory response, as evidenced not only by an accompanying elevation in inflammatory markers, including C-reactive protein (CRP) and D-dimer [8, 9], but also by the detection of inflammatory cells such as lymphocytes and macrophages in aortic media [7, 10]. Toll-like receptor 4 (TLR4) is one of the most well-characterized inflammation-related molecules, which can recognize both pathogens and endogenous ligands and initiate inflammatory reactions [11]. Immune inflammatory cells are primary sources of TLR4. In addition, TLR4 can be expressed in vascular-related cells, particularly endothelial cells and smooth muscle cells, and participate in mediating vascular inflammation and remodeling [12, 13]. Recently, TLR4 role has emerged in maintaining aortic homeostasis and evoking aortic aging and diseases [14]. Therefore, it is reasonable to suppose that TLR4 may play a crucial role in pathogenesis of AAD. However, to date, no researcher has tried to discuss the association of serum TLR4 levels with AAD. In addition, current data have reported the role of TLR4-mediated signaling pathway in triggering inflammatory cell infiltration and media degeneration in the aortic wall, and regulating MMP9 expression in human aortic smooth muscle cells [15–17]. However, to our knowledge, there was lacking evidence concerning the relationship between serum MMP9 and TLR4 expression.

In this study, we evaluated serum MMP9 and TLR4 levels in AAD patients and healthy controls. Then, we investigated the possible association between serum MMP9 and TLR4, and their correlation with classical biomarkers of AAD, such as CRP and D-dimer. Furthermore, we attempted to clarify MMP9 and TLR4 alone, and their combined diagnostic efficacy for AAD detection.

## Methods

### Study population

A total of 88 patients diagnosed as AAD and 88 age and gender matched healthy controls were enrolled in this study from the First Hospital of China Medical University between March 2017 and January 2018. The diagnosis of all patients who were admitted to our emergency department for evaluation within 24 h after symptom onset was confirmed by computed tomography angiography (CTA). AAD patients were then classified in Stanford type A (*n* = 47) and type B (*n* = 41). The exclusion criteria included subjects with chronic aortic dissection, coronary heart disease, congenital heart disease, severe vascular stenosis, autoimmune diseases, severe organ failure, infectious diseases, malignant tumors, hematological system diseases, previous aortic surgery or received non-steroidal anti-inflammatory drugs or steroids. The demographic data, risk factors and clinical test related information including CRP and D-dimer levels were collected from patients’ electronic medical records. The tests used for CRP and D-dimer evaluation were according to our hospital laboratory uniform measurement standards and methods. BC-5390CRP blood cell analyzer (Mindray) and matching reagents were used for CRP evaluation, and Immuno-Turbidimetric Assay of D-Dimer (STA-LIATEST® D-DI) was used for D-dimer detection. This study was approved by the Ethics Committee of the First Hospital of China Medical University (Shenyang, China). Written informed consent was obtained from each subject.

### Detection of serum TLR4 and MMP9

Approximately 5 ml fasting blood sample was collected from each participant using standardized sterile tubes. All samples were centrifuged immediately at 3500 r/min for 10 min at 4 °C, and the serum was separated, and stored at − 80 °C until analysis. Serum MMP9 and TLR4 levels were measured by ELISA using ELISA kits (MMP9: Wuhan Boster Biotechnology Company, Wuhan, Hubei, China; TLR4: CUSABIO Company, Wuhan, Hubei, China), according to the manufacturer’s protocol.

### Statistical analysis

Statistical analyses were performed using SPSS 17.0 and STATA 11.0 software. Continuous variables were reported as mean values and standard deviations, and categorical variables were represented as numbers and percentages. Statistical tests used in the study were independent-sample t-test, Chi-squared test, Mann-Whitney U-test and Spearman correlation as appropriate. Multiple logistic regression models were performed to assess the correlation of serum MMP9 and TLR4 with AAD risk after adjusting the potential confounding factors. Receiver operator characteristic (ROC) curves with area under the curve (AUC) based on logistic models were used to determine the corresponding cut-off points and measure the diagnostic performance of serum MMP9 and TLR4 individually, and combined for AAD detection. A *p* value≤0.05 (two-sided) was considered statistically significant.

## Results

### Baseline characteristics of the study participants

The detailed clinical data of the cases and controls are presented in Table 1. Compared to control group, subjects in overall AAD, type A and type B groups tended to have significantly higher levels of heart rate, white blood cell (WBC), creatinine and homocysteine (Hcy), and higher rates of hypertension and diabetes, but lower level of hemoglobin (Hb). Creatinine and D-dimer levels were lower in type B group compared with type A group. In addition, there were no significant differences in other comparisons between two types of AAD.

**Table 1:** Clinical characteristics of the study subjects

Variables	Control(*n* = 88)	AAD(*n* = 88)	Type A(*n* = 47)	Type B(*n* = 41)
Age, years	58.6 ± 12.1	56.7 ± 12.4	56.3 ± 10.7	57.2 ± 14.1
Males, n (%)	65(73.9%)	64(72.7%)	36(76.6%)	28(68.3%)
Smoking, n (%)	35(40.0%)	33(37.5%)	17(36.2%)	16(39.0%)
Drinking, n (%)	37(42.0%)	31(35.2%)	18(38.3%)	13(31.7%)
BMI, kg/m^2^	25.51 ± 3.25	25.38 ± 5.69	24.91 ± 6.72	25.94 ± 4.18
Heart rate, bmp	73.91 ± 9.68	78.51 ± 14.42^*^	78.09 ± 15.60^*^	78.97 ± 13.15^*^
WBC, 10^9^/L	5.79 ± 1.85	11.51 ± 4.48^*^	12.33 ± 4.45^*^	10.58 ± 4.39^*^
Hb, g/L	150.81 ± 13.19	132.49 ± 20.93^*^	132.13 ± 20.34^*^	132.90 ± 21.83^*^
Hypertension, n (%)	40(45.5%)	64(72.7%)^*^	34(72.3%)^*^	30(73.2%)^*^
Diabetes, n (%)	11(12.5%)	31(35.2%)^*^	18(38.3%)^*^	13(31.7%)^*^
Hyperlipidemia, n (%)	23(26.1%)	22(25.0%)	12(25.5%)	10(24.4%)
Creatinine, umol/L	66.10 ± 13.55	77.96 ± 22.99^*^	92.48 ± 35.91^*^	69.83 ± 18.38^#^
Hcy, umol/L	10.91 ± 3.31	15.69 ± 7.33^*^	15.36 ± 11.10^*^	15.79 ± 6.15^*^
CRP, mg/L	–	75.58 ± 54.37	79.03 ± 55.17	71.69 ± 53.89
D-dimer, ug/ml	–	7.13 ± 6.88	8.98 ± 7.74	5.38 ± 5.50^#^

^*^p < 0.05 vs. control, ^#^*p* < 0.05 vs. Type A

### Serum MMP9 and TLR4 levels in AAD

Serum MMP9 and TLR4 levels of AAD patients and controls are reported in Table 2. Overall AAD, type A and type B patients all showed significantly higher MMP9 and TLR4 levels than those in control group. However, no significant differences in MMP9 and TLR4 levels were observed between type A and type B patients.

**Table 2:** Serum levels of MMP9 and TLR4 in different groups

Variables	Control(*n* = 88)	AAD(*n* = 88)	Type A(*n* = 47)	Type B(*n* = 41)
MMP9, ng/ml	229.01 ± 137.93	364.00 ± 117.52^*^	358.35 ± 131.26^*^	370.48 ± 100.71^*^
TLR4, ng/ml	6.29 ± 3.21	13.07 ± 9.88^*^	12.13 ± 9.21^*^	14.15 ± 10.61^*^

^*^p < 0.05 vs. control

Furthermore, we separately compared MMP9 and TLR4 levels between AAD and control subjects stratified by cardiovascular risk factors (Table 3). AAD patients had much higher levels of either MMP9 or TLR4 in each stratified analysis. In addition, we performed multiple logistic regressions to evaluate the predictive value of serum MMP9 and TLR4 in AAD with the adjustment for age, gender, smoking, drinking, hypertension, diabetes and hyperlipidemia. There was an independent significant association of AAD risk with serum MMP9 (OR = 1.010 per unit increase, 95% CI = 1.006–1.013, *p* < 0.001) and TLR4 (OR = 1.393 per unit increase, 95% CI = 1.232–1.576, *p* < 0.001).

**Table 3:** Comparison of serum MMP9 and TLR4 levels between AAD and control group stratified by cardiovascular risk factors

Variables		Control	AAD		Control	AAD	
		MMP9(ng/ml)	MMP9(ng/ml)	*p* vs. Control	TLR4(ng/ml)	TLR4(ng/ml)	*p* vs. Control
Age	<60y	143.91 ± 117.89	378.40 ± 92.86	< 0.001	4.43 ± 2.67	12.39 ± 9.89	< 0.001
	≥60y	299.93 ± 111.27	348.23 ± 139.12	0.008	7.84 ± 2.78	13.81 ± 9.94	0.001
Gender	male	233.88 ± 140.40	364.87 ± 121.92	< 0.001	6.18 ± 3.30	13.74 ± 11.17	< 0.001
	female	215.25 ± 132.76	361.55 ± 106.59	< 0.001	6.61 ± 2.99	11.16 ± 4.27	< 0.001
Smoking	Yes	218.50 ± 143.35	354.69 ± 121.82	< 0.001	6.04 ± 2.64	14.68 ± 11.66	< 0.001
	No	235.95 ± 135.17	369.87 ± 115.49	< 0.001	6.45 ± 3.55	12.05 ± 8.54	< 0.001
Drinking	Yes	232.46 ± 144.07	361.91 ± 121.77	< 0.001	6.11 ± 2.99	12.42 ± 10.17	0.002
	No	226.51 ± 134.70	365.14 ± 116.22	< 0.001	6.42 ± 3.38	13.42 ± 9.79	< 0.001
Hypertension	Yes	245.21 ± 131.57	348.85 ± 123.60	< 0.001	6.93 ± 3.54	11.24 ± 6.92	< 0.001
	No	215.52 ± 142.98	404.40 ± 89.71	< 0.001	5.75 ± 2.83	17.95 ± 14.28	< 0.001
Diabetes	Yes	207.47 ± 147.81	360.06 ± 126.11	0.003	7.66 ± 4.05	13.76 ± 11.84	0.019
	No	231.77 ± 137.38	366.15 ± 113.67	< 0.001	6.11 ± 3.07	12.69 ± 8.73	< 0.001
Hyperlipidemia	Yes	272.67 ± 130.16	404.97 ± 87.75	< 0.001	6.99 ± 3.12	15.56 ± 9.49	< 0.001
	No	213.57 ± 138.25	350.34 ± 123.45	< 0.001	6.04 ± 3.23	12.24 ± 9.94	< 0.001

### Correlations of serum MMP9 and TLR4 with CRP and D-dimer

**Fig. 1:**
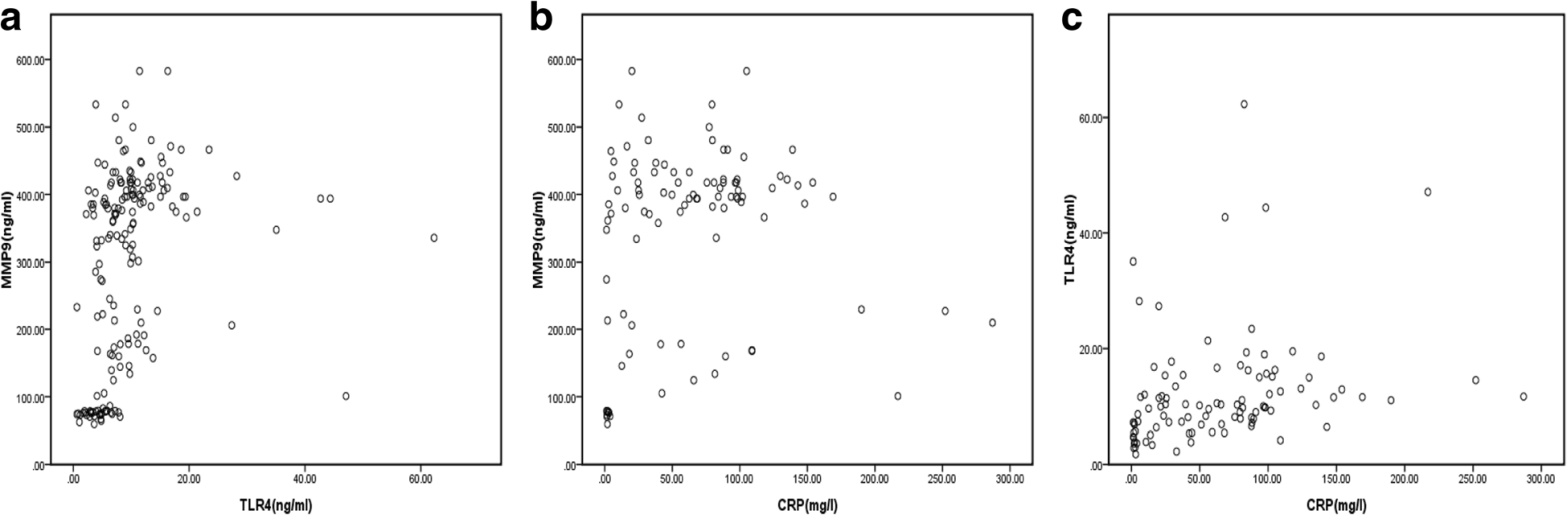
Relationship between serum MMP9 and TLR4 (**a**) and the correlation of CRP with MMP9 (**b**) and TLR4 (**c**)

We further assessed the possible relationship between serum MMP9 and TLR4, and their correlations with CRP and D-dimer. The results showed that there was a statistically significant association between serum MMP9 and TLR4 (*r* = 0.518, *p* < 0.001) (Fig. 1). Moreover, CRP was positively correlated with both MMP9 (*r* = 0.237, *p* = 0.019) and TLR4 (*r* = 0.436, *p* < 0.001) (Fig. 1). However, D-dimer was associated with neither MMP9 (*r* = 0.157, *p* = 0.170) nor TLR4 (*r* = 0.124, *p* = 0.281).

### Diagnostic performance of serum MMP9 and TLR4 and their combination for AAD

**Fig. 2:**
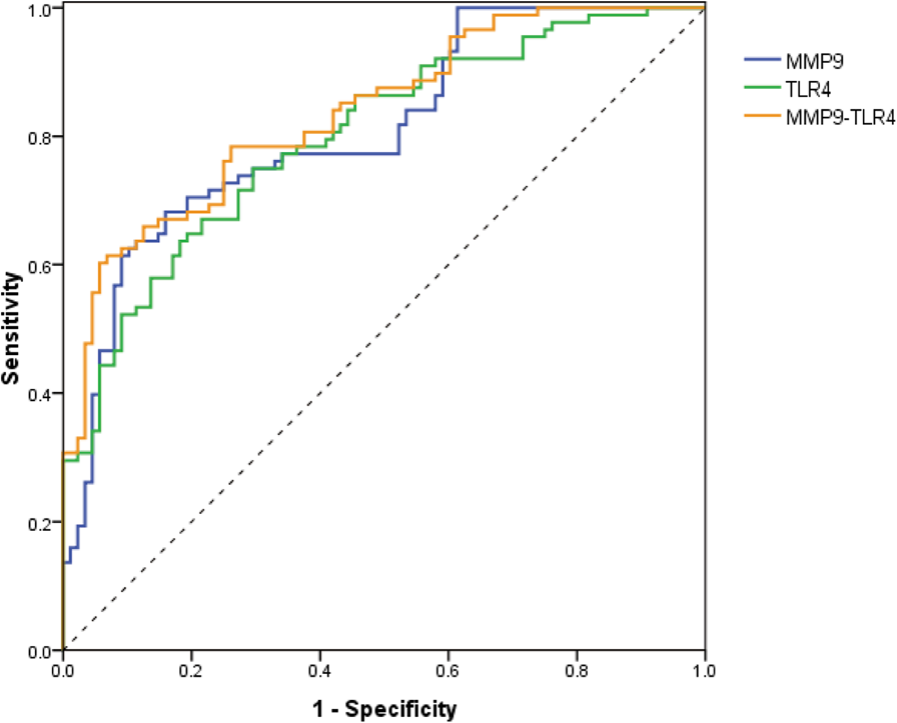
ROC curves for serum MMP9 and TLR4 individually and combined for identifying AAD

Based on ROC analysis, diagnostic performance of serum MMP9 and TLR4 and their combination in discriminating AAD are shown in Table 4 and Fig. 2. The AUCs for MMP9 and TLR4 alone were 0.810 and 0.799 with corresponding optimal cut-off points of 379.47 ng/ml and 7.83 ng/ml associated with a sensitivity of 68.2% and 75.0%, and a specificity of 84.1% and 70.5%, respectively. For their combined model (MMP9-TLR4), the AUC (0.837) was significantly greater than TLR4 (*p* = 0.046) alone, but not MMP9 (*p* = 0.230). Furthermore, MMP9-TLR4 yielded a sensitivity of 60.2% and specificity of 94.3% at the predicted probability of 0.68 as the optimal cut-off point.

**Table 4:** Accuracy of serum MMP9 and TLR4 alone and their combination for AAD detection

	Cut-off value	AUC	95% CI	Sensitivity	Specificity	YD	*p*-Value
MMP9, ng/ml	379.47	0.810	0746–0.873	68.2%	84.1%	0.523	< 0.001
TLR4, ng/ml	7.83	0.799	0.735–0.863	75.0%	70.5%	0.455	< 0.001
MMP9-TLR4	0.68	0.837^*^	0.779-0.894	60.2%	94.3%	0.545	< 0.001

^*^p < 0.05 vs. TLR4

## Discussion

As two important members closely related to AAD, the role of both MMP9 and TLR4 is not to be underestimated. Serum MMP9 represents the leakage of enzyme into the bloodstream during periods of matrix catabolism and its elevation may reflect a more active state of degeneration of the aortic wall in the natural history of aortic dissection [6]. The circulating form of TLR4 is produced from the conversion of cell-surface TLR4 and believed to have a similar immuno-inflammatory regulation role. Serum TLR4 has been reported to be elevated as a potential biomarker that provides information about the systemic status associated with inflammatory conditions [18–20]. In the present study, to the best of our knowledge, we reported for the first time that either MMP9 or TLR4 levels were significantly increased in the serum of AAD patients not only in the overall comparison but also in subgroup analysis stratified by cardiovascular risk factors, which suggested that both MMP9 and TLR4 could be potential biomarkers to distinguish between normal aorta and AAD. According to the multiple logistic regressions, we furtherly demonstrated that either serum MMP9 or TLR4 had the predictive value in AAD risk with the adjustment for the possible confounding factors.

Here are some possible mechanisms underlying the increased serum levels of MMP9 and TLR4. Previous studies showed that overexpression of MMP9 could induce pathological changes in aortic wall, including extracellular matrix degradation, and smooth muscle cells apoptosis and phenotypic transition, which were responsible for the instability of aortic media and the occurrence of AAD [21, 22]. It has also been demonstrated that an inflammatory reaction can destroy the medial layer of the aorta and eventually lead to dilation, dissection and rupture of aortic wall [23, 24]. Moreover, local inflammation in aortic wall and systemic inflammatory responses were observed during the whole course of AAD [9, 10, 25]. TLR4 is a useful marker for evaluating inflammatory response and vascular injury, and its activation plays a critical role in vascular remodeling [12–14]. Therefore, TLR4 attracted our particular interest and we supposed that TLR4 might be important in the initiation and progression of AAD. Interestingly, our research preliminary indicated the possible association of higher circulating TLR4 levels with AAD. However, no significant differences in MMP9 and TLR4 levels were found between type A and type B AAD, indicating that serum MMP9 and TLR4 did not correlate with the specific type of AAD.

Another important finding of the present study was that serum levels of MMP9 and TLR4 were positively correlated. Data from current experiments demonstrated that the activation of TLR4-mediated signaling could induce MMP9 secretion in a variety of cells, such as vascular smooth muscle cells, macrophages and neutrophils [16, 17, 26], in contrast, lack of TLR4 function attenuated MMP9 expression [27]. During aortic tissue damage and remodeling process, released fragments from extracellular matrix degradation are known to trigger TLR4 and activate its downstream signaling [28]. These evidences partly explained the close relation between serum MMP9 and TLR4 in our study, indicating the crucial role of their interaction in the pathogenesis of AAD.

Nowadays, elevated circulating biomarkers of vascular damage, inflammation and thrombosis, such as CRP and D-dimer, have been demonstrated to be associated with AAD [29, 30]. CRP is a sensitive acute phase reactant widely used in daily routine clinical practice and has become an independent predictor of poor prognosis in AAD patients [8, 31], but can hardly be considered as a diagnostic biomarker of AAD [32]. Data has demonstrated that CRP can induce MMP9 production in human mononuclear cells in a concentration-dependent manner [33]. Moreover, CRP is not only an inflammatory marker but also a trigger that provokes inflammatory response by activating TLR4 [34]. On the other hand, TLR4 may also contribute to the enhanced pro-inflammatory effect of CRP in the vessel [35]. D-dimer, a kind of degradation product of cross-linked fibrin detectable in the serum following thrombus fibrinolysis, can provide valuable diagnostic and prognostic information for AAD patients [32, 36]. A meta-analysis demonstrated that D-dimer was a useful screening tool for excluding AAD [37]. Alexander et al. found no significant relationship between MMP9 and D-dimer in arthroplasty patients [38], while Pastorelli et al. showed a positive correlation of serum TLR4 with D-dimer in inflammatory bowel diseases [39]. Moreover, Giachino et al. proved that serum MMP9 was significantly correlated with both CRP and D-dimer in patients with AAD [40]. However, there was no evidence concerning the association of serum TLR4 with CRP or D-dimer in AAD. Preliminarily, our results indicated that both serum MMP9 and TLR4 were significantly related to CRP, which may reflect their possible interactive inflammatory reaction on aortic damage in AAD patients. However, there was no obvious relationship of serum MMP9 or TLR4 with D-dimer in AAD, which may contribute to the heterogeneity and sample size of different diseases.

Based on mostly elevated serum levels of MMP9 and TLR4 in AAD patients, we furtherly evaluated the diagnostic value of serum MMP9 and TLR4 alone and their combination for AAD detection, which may be helpful for making a reasonable clinical decision in diagnostic and screening procedure. On the basis of ROC curves, our data revealed that MMP9 had a relatively high sensitivity and specificity for the diagnosis of AAD, while TLR4 could provide a better sensitivity but relatively lower specificity. Of note, the negative predictive value of TLR4 increased from 70.5% when used alone to 94.3% when used in association with MMP9. Interestingly, MMP9-TLR4 improved the diagnostic accuracy with a significantly higher AUC than TLR4 alone for identifying AAD. These findings can provide the clues that serum MMP9 and TLR4 may be valuable non-invasive markers with potential predictive features for AAD and a combined use of them represents a promising strategy to safely rule out AAD. However, it should point that, in our study, about a quarter of AAD patients had serum MMP9 and TLR4 levels lower than the cut-off values, which might be associated with individual differences in reactivity. In addition, although strict exclusion criteria have been applied, there were still uncontrollable factors and potential variations that might influence serum levels of above two indicators.

This study has some limitations. First, this was a single-center study limited to a specific study population. Second, dynamic changes of serum MMP9 and TLR4 levels were not measured. Third, there was a lack of information of circulating CRP and D-dimer levels in control group. In addition, the sample size was relatively small. In the future, a large prospective study should be performed to confirm the diagnostic efficacy and accuracy of this novel assay. Moreover, combining tissue expression and mechanism research will give more detailed information regarding the role of MMP9 and TLR4 in AAD.

## Conclusions

Our results showed that both serum MMP9 and TLR4 levels were significantly increased in AAD patients. Then, we suggested a close association between MMP9 and TLR4 and their individual positive correlations with CRP at the level of serology. Furthermore, we provided the proof that either MMP9 or TLR4 alone may be a useful biomarker for AAD identification, but the combination of both markers could improve diagnostic performance with the potential to safely rule out AAD.

## Abbreviations

AAD: Acute aortic dissection
AUC: Area under the curve
BMI: Body mass index
CRP: C-reactive protein
CTA: Computed tomography angiography
ELISA: Enzyme-linked immunosorbent assay
Hb: Hemoglobin
Hcy: Homocysteine
MMP9: Matrix metalloproteinase 9
ROC: Receiver operating characteristic
TLR4: Toll-like receptor 4
WBC: White blood cell
